# The Role of Macrophage Autophagy in Asthma: A Novel Therapeutic Strategy

**DOI:** 10.1155/2023/7529685

**Published:** 2023-05-04

**Authors:** Lijie Wang, Xingxing Yuan, Zhuying Li, Fumin Zhi

**Affiliations:** ^1^Department of Respiratory Medicine, The First Affiliated Hospital, Heilongjiang University of Chinese Medicine, Harbin 150040, China; ^2^Heilongjiang University of Chinese Medicine, Harbin 150040, China; ^3^Department of Gastroenterology, Heilongjiang Academy of Traditional Chinese Medicine, Harbin 150006, China; ^4^Department of Medical, The First Affiliated Hospital, Heilongjiang University of Chinese Medicine, Harbin 150040, China

## Abstract

Asthma is a chronic respiratory disease frequently associated with airway inflammation and remodeling. The development of asthma involves various inflammatory phenotypes that impact therapeutic effects, and macrophages are master innate immune cells in the airway that exert diverse functions including phagocytosis, antigen presentation, and pathogen clearance, playing an important role in the pathogeneses of asthma. Recent studies have indicated that autophagy of macrophages affects polarization of phenotype and regulation of inflammation, which implies that regulating autophagy of macrophages may be a potential strategy for the treatment of asthma. Thus, this review summarizes the signaling pathways and effects of macrophage autophagy in asthma, which will provide a tactic for the development of novel targets for the treatment of this disease.

## 1. Introduction

Asthma is a heterogeneous disease of the airways with many respiratory symptoms that are characterized by airway inflammation, bronchial hyperresponsiveness, reversible airflow obstruction, and airway wall remodeling [1]. Chronic airway inflammation is common and heterogeneous in asthmatic patients, and its controlling is the key to developing effective and efficient personalized treatments [2]. Emerging biological therapies targeting airway inflammation in preclinical studies have been illustrated to improve asthmatic symptoms but provide limited benefits for patients with severe asthma; moreover, patients with different asthma inflammatory phenotypes have distinct treatment responses [3]. Thus, it is particularly crucial to seek effective therapeutic approaches for the management of asthma.

Autophagy is a cellular process that can degrade and recycle damaged organelles, misfolded protein aggregates, and other toxic substances in autolysosomes, playing an important role in maintaining metabolic homeostasis within cells [4]. Autophagy has been implicated in the development and progression of asthma and displays both protective and detrimental roles in allergic airway inflammation and remodeling [5]. Therefore, regulation of autophagy may provide a promising therapeutic strategy for this disease. Furthermore, autophagy is regarded as a vital defense mechanism to protect the body against external pathogens and is suggested to be involved in the induction and regulation of inflammatory reactions in the innate immunity [6]. Macrophages are innate immune cells and serve a critical role in immune surveillance and the development of innate and adaptive immune responses in asthma [7]. Macrophage autophagy is associated with phagocytosis, antigen presentation, and pathogen clearance [8]. Asthma-mediated autophagy in macrophages also affects its own phenotypic changes and effector functions, alleviating airway inflammatory responses or aggravating tissue damage [9, 10]. Unfortunately, the mechanism of macrophage autophagy in asthma is still unclear, and its effect on disease progression is still controversial. This review concisely summarizes the role of macrophage and autophagy in asthma and emphasizes the research progression on the autophagy-related signaling pathways in macrophages and roles of macrophage autophagy in asthma, as well as discusses the therapy potential of regulating macrophage autophagy for this disease.

## 2. Macrophages and Asthma

**Figure 1:**
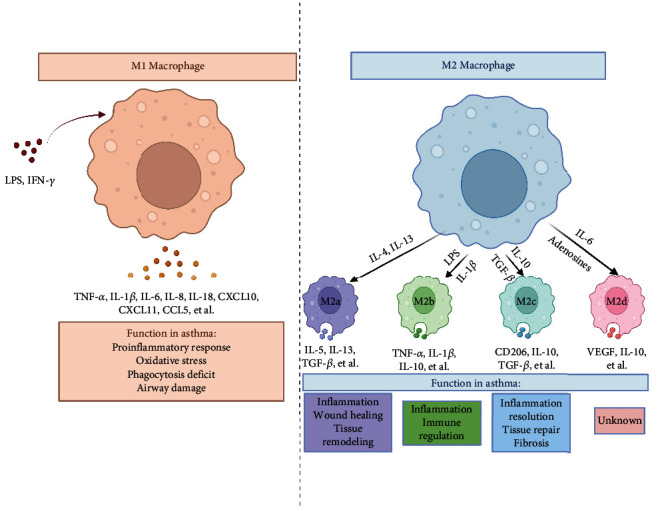
Role of macrophages in asthma. Macrophages can be polarized into the M1 phenotype by LPS or IFN-*γ* stimulation and secrete inflammatory mediators to exert a proinflammatory effect; moreover, they can also be transformed into the M2 phenotype with different subtypes (M2a, M2b, M2c, and M2d) activated by IL-4, IL-10, IL-13, etc. and have a distinct biological function. LPS: lipopolysaccharide; IL: interleukin; IFN: interferon; TNF: tumor necrosis factor; TGF: transforming growth factor; CCL: CC-motif chemokine ligand; VEGF: vascular endothelial growth factor.

As one of the master immune cells, macrophages are activated to elicit the innate immune reaction in response to inflammatory stimulus and possess diverse functions including phagocytosis, antigen presentation, inflammation induction, and pathogen clearance [7]. In general, there are two phenotypes of macrophage polarization, including M1 and M2 phenotypes; functionally, M1 macrophages have a proinflammatory effect, while M2 macrophages exert an immunomodulatory effect with various subtypes, as described in (Figure 1) [11].

M1 macrophages can be activated by several stimulators, such as lipopolysaccharide (LPS) and interferon (IFN)-*γ* and subsequently secrete amounts of proinflammatory cytokines (e.g., tumor necrosis factor- (TNF-) *α*, interleukin- (IL-) 1*β*, and IL-6), and chemokines (e.g., CC-motif chemokine ligand (CCL)2, CCL5), which play a crucial role in the pathogen phagocytosis, antigen recognition, and the initiation of adaptive immune responses [12]. During asthmatic inflammation, macrophages are recruited into the airway tissue and bronchial epithelium and release high levels of inflammatory mediators, contributing to chronic airway inflammation [13]. Among them, TNF-*α*, CCL-17, and IL-6 are required for the constitution of innate memory in allergen-triggered inflammation, which may perpetuate and exacerbate airway inflammation [14]. The activation of the NOD-like receptor protein (NLRP) 3 inflammasome also contributes to a proinflammatory phenotype in macrophages upon respiratory viral infection, which is correlated with asthma severity [15, 16]. Both inflammation and oxidative stress affect macrophage function, which may result in exaggerated asthmatic damage in the airways [17]. Besides, macrophages from patients with asthma display lower phagocytic capacity of bacteria, yeasts and particulate matter than macrophages from controls, suggesting that impaired macrophage phagocytosis is associated with the progression of asthma [18]. These findings suggest that excessive production of proinflammatory mediators, activation of the NLRP3 inflammasome, and deficit of phagocytosis in macrophages participate in the development of asthma.

Based on the induced stimuli and phenotypic features, M2 macrophages are categorized into four distinct subtypes, namely, M2a, M2b, M2c, and M2d [12]. These macrophages vary in their expressed markers, secreted cytokines, and biological functions. M2a macrophages are activated by IL-4, IL-13, and fungal infections, expressing high levels of CD206, ARG1, YM1, FIZZ1, and TGF-*β*, resulting in allergic inflammation and wound healing. M2b macrophages are stimulated by LPS, IL-1*β*, and immune complex and release proinflammatory cytokines including IL-1*β*, IL-6, and TNF-*α*, and anti-inflammatory IL-10, acting as immunoregulatory macrophages. M2c macrophages are elicited by IL-10, glucocorticoids, and TGF-*β*, expressing high levels of innate receptors CD206 and CD163, and are essential for the tissue remodeling and fibrosis. M2d macrophages are induced by IL-6 and adenosines, which are highly expressed vascular endothelial growth factor, and IL-10, participating in angiogenesis and tumor growth [19, 20]. The anti-inflammatory behavior of M2 macrophages is largely attributed to the release of IL-10, since a lack of IL-10 in macrophages contributes to the pathogenesis of asthma; moreover, supplementing with IL-10 causes less inflammation in airway [21]. Also, expanding the lung IL-10-producing macrophages through intranasal delivery of mesenchymal stem cell-derived exosomes relieves the symptoms of allergic asthma [22]. Macrophages play a vital role in repair and remodeling via the removal of damaged tissue, thereby mediating tissue repair. For instance, macrophage efferocytosis, also known as the ingestion of apoptotic cells, facilitates the resolution of allergic airway inflammation [23]. Therefore, it can be concluded that controlling excessive inflammatory responses and improving anti-inflammatory functions, by regulating the function of macrophages, will provide promising therapeutic targets for asthma.

## 3. Autophagy and Asthma

**Figure 2:**
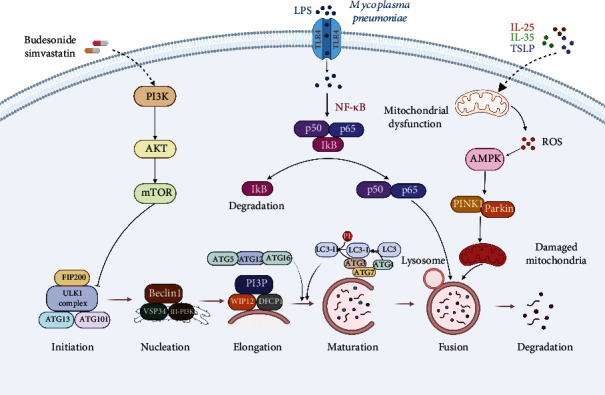
Mechanisms of macrophage autophagy in asthma. Signaling pathways including the PI3K/AKT/mTOR, TLR4/NF-*κ*B, and AMPK are involved in the regulation of macrophage autophagy in asthma. AMPK: adenosine monophosphate-activated protein kinase; PI3K: class III phosphatidylinositol 3-kinase; ATG: autophagy-related gene; ROS: reactive oxygen species; TLR, toll-like receptor; LC3: microtubule associated protein light chain 3; mTOR: mammalian target of rapamycin; VPS15: vacuolar protein sorting 15; WIPI2: WD repeat domain phosphoinositide-interacting protein; TSLP: lymphopoietin.

The process of autophagy contains initiation, nucleation, elongation, maturation, fusion, and degradation, all of which are regulated by a variety of autophagy-related genes (ATG) and signaling pathways (Figure 2) [24]. Initially, unc-51 like autophagy activating kinase (ULK1), ATG17, ATG13, and ATG101 form a protein complex named a preautophagosomal structure (PAS) [24]. Subsequently, the autophagy and beclin-1 regulator (AMBRA) protein is phosphorylated to activate the class III phosphatidylinositol 3-kinase (PI3K) complex, which is composed of PI3KC3, Beclin1, ATG14, and vacuolar protein sorting 15 (VPS15), producing phosphatidylinositol 3-phosphate (PI3P) [25]. Next, zinc-finger FYVE domain-containing protein 1 (ZFYVE1, also called DFCP1) and WD repeat domain phosphoinositide-interacting protein (WIPI2) are incorporated into PI3P, which binds the ATG16L1 and further recruits the ATG16L1/ATG5/ATG12 protein complex into the PAS. This complex also combines with the ATG3/ATG7 complex to facilitate the binding of microtubule-associated protein light chain 3 (LC3)-II, a catalysate from LC3 by the cysteine protease ATG4, with phosphatidylethanolamine (PE) for the formation of a mature autophagosome, and further to interact with a cargo receptor p62 to target proteins or organelles [26]. Finally, the autophagosome fuses with the lysosome to form the autolysosome. As the fusion completes, the autophagosome substances will be degraded and released from the autolysosome [27]. These products of decomposition can be recycled for cellular anabolism and growth. As the central step to regulate autophagy, the mammalian target of rapamycin (mTOR) signaling pathway can be further modulated by various signaling pathways, such as PI3K/AKT, adenosine monophosphate-activated protein kinase (AMPK), and mitogen-activated kinase-like protein (MAPK) [28].

Studies has demonstrated that genetic polymorphisms of the ATG5 in patients with asthma are related to airway remodeling and impairment [29]. Also, peripheral blood cells from asthmatic patients have elevated LC3-II levels compared to healthy controls [30, 31]. These findings suggest autophagy is activated in asthma and has a relationship with disease progression. Experimentally, in the ovalbumin-induced allergic airway inflammation mouse model, the expression levels of ATG5, LC3-II, and Beclin1 in macrophages from lung homogenates and bronchoalveolar lavage fluid are decreased; moreover, these autophagy proteins are enhanced after administration with simvastatin, which is associated with attenuated inflammatory cytokine production, airway inflammation, and remodeling [32]. Besides, in ATG5-deficient mice, lack of autophagy causes neutrophilic airway inflammation and hyperreactivity, concomitant with increased IL-1*β* and IL-17 levels in lung lysates upon house dust mite sensitization and challenge [33]. Likewise, enhanced eosinophilic inflammation and airway hyperreactivity are also observed in ATG5-deficient obese mice compared to wild type obese mice, suggesting that autophagy mitigates the exacerbation of eosinophilic inflammation in obese asthma [34]. Intriguingly, the level of autophagy differs in distinct cell types in asthma-like animal models. It is reported that the expression of p62 is upregulated in splenocytes but decreased in the neutrophils extracted from the bronchoalveolar fluid in chronic house dust mite-induced airway inflammation; moreover, targeting autophagy by the therapeutic peptide P140 restores the basal level of autophagy in splenocytes and enhances autophagy in neutrophils, attenuating allergic airway inflammation, suggesting a protective effect of autophagy in neutrophils [35]. Thus, autophagy plays a protective role by regulating the inflammatory response and alleviating allergic airway inflammation and remodeling.

However, autophagy exacerbates asthmatic airway inflammation and generates a detrimental effect on patients with asthma. For example, deficient autophagic flux and elevated p62 expression are detected in lung homogenates, which are linked to exacerbated airway remodeling in a chronic asthma model [36]. In addition, the administration of yeast fermentate prebiotic suppresses perivascular infiltrations of eosinophils and ameliorates oxidative damage to the lung of asthmatic mice, associated with enhanced expression of ATG5, Beclin1, and LC3-II [37]. Similarly, *α*1-antitrypsin exerts antiasthmatic effects by alleviating airway allergic inflammation and oxidative stress through suppressing autophagy and thus ameliorating asthma [38]. In support, increased levels of IL-4, IL-5, and IL-13 in bronchoalveolar lavage fluid, infiltration of inflammatory cells in lung tissues, and airway hyperresponsiveness are observed along with autophagy activation in ovalbumin-induced mice; also, these effects are inhibited by luteolin, which blocks autophagy by activating the PI3K/AKT/mTOR pathway and suppressing the Beclin1-PI3KC3 protein complex [39].

## 4. Signaling Pathways of Macrophage Autophagy in Asthma

### 4.1. The AMPK Signaling Pathway

The MAPK signaling pathway exerts vital functions in the regulation of cell proliferation, differentiation, apoptosis, and inflammation; it is also involved in the progression of asthma [40, 41]. As the main energy-sensing kinase, MAPK is activated to restore energy homeostasis and coordinate mitochondrial metabolism in response to rising AMP levels following ATP hydrolysis [42]. In impaired mitochondria, excessive reactive oxygen species (ROS) accumulation derived from mitochondrial complexes can activate AMPK, which directly phosphorylates mitochondrial fission factor to regulate mitochondrial fission and stimulate ULK1, the upstream kinase in autophagy, thus leading to mitophagy [43]. Several studies have shown that treatment with the lymphopoietin, IL-25, and IL-33, which are secreted from airway epithelial cells in asthma, induces mitochondrial ROS production, and increases the expressions of mitophagy-related proteins PINK1, Parkin, and LC3 in macrophages, suggesting that mitophagy occurs in macrophages, which contributes to M2 macrophage polarization in monocytes [44–46]. This enhanced expression of PINK1. Parkin and LC3 are reduced by dorsomorphin, an AMPK inhibitor, suggesting that autophagy is induced in macrophages by activating the MAPK signaling pathway [45, 46]. Therefore, these results suggest that under stressed conditions, dysfunctional mitochondria in macrophages cause excessive ROS accumulation and further induce mitophagy by activating the MAPK signaling pathway, which may serve as an adaptive response to alleviate oxidative stress and inflammatory responses in asthma.

### 4.2. The TLR4 Signaling Pathway

As an important pathogenic factor in asthma, the *mycoplasma pneumoniae* infection has been demonstrated to induce macrophage autophagy through toll-like receptor (TLR)4-dependent pathways [9, 47]. In TLR2 KO macrophages infected with *mycoplasma pneumoniae*, the cytokines and inflammatory responses are increased, and these effects are abrogated by both autophagy inhibitors and TLR4 knockdown, implying that *mycoplasma pneumoniae* triggers inflammatory responses in macrophages through TLR4 and autophagy [47]. Further mechanistic investigation revealed that in response to *mycoplasma pneumoniae*, the macrophage inflammation is initiated by the TLR4 signaling pathway, which subsequently establishes a positive feedback loop between the NF-*κ*B signaling cascade and autophagy and further activates the NLRP3 inflammasome, ultimately inducing TNF-*α* and IL-1*β* production [9]. LPS, a TLR4 ligand, is regarded as an exacerbating factor in severe asthma and induces macrophage autophagy through the downstream toll-interleukin-1 receptor domain-containing adaptor-inducing interferon-*β* (TRIF)/p38 MAPK signaling pathway [48]. The activated TLR4 also suppresses the interaction of Beclin1 with Bcl-2 by recruiting Beclin1 into the TLR4/MyD88 (myeloid differentiation factor 88) complex, leading to macrophage autophagy [49]. These results indicate that inflammatory stimulus promote macrophage autophagy through the TLR4 signaling pathway. Thus, targeting TLR4 through Hirsutella sinensis mycelium, a natural agent, reduces the cumulation of autophagosomes, and achieves anti-inflammation effects [50].

### 4.3. The mTOR Signaling Pathway

The mTOR signaling pathway participates in the regulation of cell growth and energy metabolism under physiological and pathological conditions; it also plays a crucial role in asthma [51]. PI3K/AKT is an important upstream regulator of the mTOR pathway. The activation of PI3K phosphorylates AKT, which subsequently stimulates the mTOR complex 1 and inactivates the tuberous sclerosis complex 2 (TSC2) to disrupt the formation of the TSC1/TSC2 heterodimer, resulting in mTORC1 activation and ultimately causing autophagy suppression [52]. It is reported that airway macrophages, obtained from asthmatic patients with inhaled budesonide treatment, present a reduced expression of Beclin1, LC3, and autophagic flux, along with increased release of IL-10, suggesting IL-10 production is associated with suppression of autophagy. Moreover, silencing of LC3 in macrophages shows a maximal induction of IL-10 transcription, and depletion of IL-10 reverses the inhibitory effects of budesonide on macrophage autophagy [10]. Further mechanistic evaluation reveals that IL-10 activates PI3K, which phosphorylates p70S6K through the activation of AKT and mTORC1 [53]. In another study, the inactivation of transcription factor EB, a major regulator of autophagy, can activate the mTORC1 signaling pathway in circulating monocytes from patients with severe asthma, and thus autophagy is impaired in this process and fails to inhibit NLRP3-driven inflammatory responses [54]. These findings indicate that the mTOR signaling pathway can serve as a regulator to affect macrophage autophagy in asthma.

## 5. Effects of Modulating Macrophage Autophagy on Asthma

### 5.1. Promote Macrophage Autophagy to Reduce Inflammation

Excessive activation of macrophages is required for the inflammatory response and cytokine release in various inflammatory diseases, including asthma. LPS is employed as an additive to mimic severe asthma and exacerbates the macrophage-induced inflammation and allergic lung damage [55]. In LPS-treated macrophages, pro-IL-1*β* is transported into autophagosomes for further autophagic degradation induced by rapamycin, an autophagy inducer, thus blocking the secretion of IL-1*β*. Moreover, macrophage autophagy also controls IL-1*β* release by suppressing of NLRP3 inflammasome activation [56]. Mitochondrial ROS is a major resource for mediating the macrophage inflammatory response in asthma. Autophagy induction by rapamycin represses the generation of IL-1*β* and IL-18 in LPS-activated macrophages through the removal of mitochondrial ROS in a p62-dependent manner; subsequently, the IL-1*β* reduction downregulates the expression of IL-6, IL-8 and monocyte chemoattractant protein 1 via inactivating of the p38 MAPK/NF-*κ*B pathway [57]. Thus, it can be speculated that macrophage autophagy impedes the production of inflammatory mediators and alleviates inflammatory responses in asthma. Activation of autophagy macrophage by sophoridine, a natural compound, alleviates LPS-mediated inflammation and acute lung injury by suppressing the TLR4/NF-*κ*B and mTOR signaling pathways [58]. Further investigation of the regulation of macrophage autophagy in asthmatic inflammation could provide a promising therapeutic strategy for this disease.

Macrophages with distinct phenotypes are detected in the airway tissue of asthmatic patients. Of the M2 macrophage subsets, M2a and M2c have been predominately implicated in allergic asthma. M2a macrophages expressing CD206 and MHCII are associated with asthma severity, and M2a macrophages expressing CCL17 and CCL22 induce the activation of T helper type 2 cells and initiate the infiltration of eosinophil in the lungs by secreting high levels of IL-5 and IL-13 [59, 60]. Moreover, during the chronic inflammation of asthma, M2a macrophages are also involved in driving tissue remodeling [61]. M2c macrophages exhibit an anti-inflammatory effect owing to greater IL-10 expression and initiate the inflammation resolution and tissue repair of airway by releasing IL-10 [62]. By producing TGF-*β* and regulating the expression of extracellular matrix molecules, they also activate fibroblasts to induce fibrosis, participating in tissue remodeling [12]. As mentioned above, M1 macrophages secrete proinflammatory factors that aggravate inflammation and tissue damage, whereas M2 macrophages release anti-inflammatory cytokines to inhibit excessive inflammation and promote tissue repair [63, 64]. However, which subtypes of M2 macrophages experience autophagy and how they function in asthma are still elusive. Recently, it has been demonstrated that autophagy triggers macrophages to polarize into the anti-inflammatory M2 phenotype. IL-33, a cytokine derived from epithelial cells, is elevated by allergens in asthma, facilitates the overproduction of ROS, and contributes to airway inflammation. In the IL-33-stimulated human monocyte cell line THP-1, at 0.5 h and 2 h of stimulation, the mitochondrial respiratory chain complex II and V and ROS level increase; after 8 h of stimulation, mitophagy-related proteins (PINK1, Parkin, and LC3) increase, which is aimed at the selective degradation of deficit mitochondria to mitigate ROS accumulation; after 24 h of stimulation, the M2 phenotype increases with enhanced expression of CCL-22, and the M1 phenotype decreases accompanied by low expression of CXCL-10 and TNF-*α*, suggesting IL-33 exerts proinflammatory and subsequent anti-inflammatory roles in asthma [44]. Likewise, IL-25 is elevated in asthma and improves mitophagy and CCL-22 secretion in monocytes; moreover, inhibition of mitophagy by PINK1 knockdown reduces the production of CCL-22. The mechanism may be that upregulation of ROS by IL-25 activates the AMPK signaling pathway and subsequently induces PINK1/Parkin-mediated mitophagy, thereby eliciting M2 macrophage polarization in monocytes and attenuating asthmatic inflammation [45]. The AMPK signaling pathway is also activated in THP-1 cells in response to thymic stromal lymphopoietin, a cytokine involved in asthma pathogenesis, and mitophagy is subsequently promoted, which results in the enhanced expression of M2-related cytokines CCL-1 and CCL-22, as well as the reduced generation of M1-related cytokine CXCL-10; moreover, these effects are interdicted by the mitophagy inhibitor Mdivi-1 [46]. In addition, macrophage exposure to acrylamide, which is associated with the occurrence and exacerbation of asthma, induces the M2-like phenotype with elevated transforming growth factor (TGF)-*β* and CCL-2 levels by activating PINK1/Parkin-mediated mitophagy, alleviating ROS-mediated proinflammatory activity [65]. These results indicate that upregulation of macrophage mitophagy can drive M2 phenotype polarization and play an anti-inflammatory role. However, these studies fail to further identify the subtypes of M2a macrophages. It should be noted that the biological function of M2 macrophages is distinct depending on their expressed markers and generated cytokines. For example, M2a macrophages expressing MRC1 and IL-1RA perform protective roles, while M2a macrophages expressing CCL17 and CCL22 exert pathogenic roles in allergic asthma [66]. In this regard, it can be speculated that the effects of autophagy in different M2 macrophage subtypes vary in this disease. Thus, future investigations should focus on the function of autophagy in various subtypes of M2 macrophage.

### 5.2. Promote Macrophage Autophagy to Activate Inflammation

Pathogen infection is believed to precede the onset of asthma or aggravate asthma symptoms. An infection with *mycoplasma pneumoniae* is positively associated with childhood asthma, and it may serve as a risk factor or contributor during asthmatic inflammatory damage [67, 68]. Several studies have reported that *mycoplasma pneumoniae* initiates inflammatory responses in macrophages through autophagy induction. One study found that in TLR2 KO macrophages infected with *mycoplasma pneumoniae*, the expression of TNF-*α* is detected at 1 h of infection and reaches a maximum level at 2 h; however, these effects are blocked by the autophagy inhibitors 3-methyladenine and chloroquine, suggesting autophagy is involved in the induction of inflammatory responses in macrophages infected with *mycoplasma pneumoniae* [68]. Further investigation unveiled that the membrane lipoprotein of *mycoplasma pneumoniae* promotes the secretion of proinflammatory cytokines TNF-*α* and IL-1*β* and the production of the NLRP3 inflammasome via activating the TLR4 signaling pathway. Deep mechanistic evaluation revealed that autophagy-induced inflammation participates in the activation of the NF-*κ*B signaling cascade and the activated NF-*κ*B accelerated lipoprotein-mediated autophagosome formation, implying a positive feedback loop between autophagy and NF-*κ*B signaling pathway, which ultimately promotes TNF-*α* and Il-1*β* generation in macrophages [9]. These findings indicate that promoting autophagy in macrophages infected with *mycoplasma pneumoniae* may contribute to inflammatory responses, expedites airway inflammation, and exacerbate asthma symptoms.

### 5.3. Inhibit Macrophage Autophagy to Alleviate Inflammation

As mentioned above, excessive macrophage autophagy deteriorates asthmatic inflammation. Consistent with this result, several studies have found that repression of macrophage autophagy ameliorates the inflammatory responses in asthma. It is reported that in airway macrophages of asthmatic patients, inhaled budesonide suppresses autophagic flux along with increased IL-10 and decreased IL-4 levels in sputum, which can be further enhanced by simvastatin [10]. In vitro experiments, after macrophages are subjected to rapamycin for autophagy induction, administration with both budesonide and simvastatin reduces the expression of Beclin1 and LC3 but increases the accumulation of p62, accompanied by enhanced expression of IL-10; moreover, silencing IL-10 transcription reverses the suppressive effects of budesonide and simvastatin on macrophage autophagy, which indicates that inhibition of macrophage autophagy by corticosteroids and a statin promote IL-10 production and thus limit asthmatic inflammation [10]. Park and et al. conducted a study on the mechanisms by which IL-10 inhibits autophagy in macrophages. They showed that IL-10 suppressed starvation-induced macrophage autophagy by inactivating the PI3K/AKT/mTOR signaling pathway [53]. In addition, the autophagy inhibitor chloroquine is demonstrated to attenuate the generation of inflammatory mediators such as NO, TNF-*α*, and IL-6 in LPS-stimulated macrophages, indicating that suppressing autophagy in macrophages exerts an anti-inflammatory effect [69]. These results reveal that inhibition of excessive macrophage autophagy may play a protective role in asthma.

### 5.4. Inhibit Macrophage Autophagy to Promote Inflammation

Exposure to particulate matter (PM), a kind of air pollutant, is associated with the development of asthma. In bronchoalveolar lavage from mice exposed to PM, the number of macrophages are increased with enhanced production of IL-1*β* and NLRP3 but reduced mitochondrial antioxidant manganese superoxide and autophagy [70]. Besides, in circulating monocytes from patients with severe asthma, impaired autophagy is concomitant with highly activated NLRP3 and mitochondrial ROS accumulation; otherwise, restoration of impaired autophagy restrains NLRP3-driven release of IL-1*β* and IL-18 and attenuates airway inflammation and severe asthma phenotypes [54]. These results suggest that the low level of macrophage autophagy is related to mitochondrial dysfunction and increased inflammation in asthma. Indeed, autophagy deficiency in macrophages is confirmed to promote inflammatory responses. For example, in response to LPS stimulation, ATG16L1-deficient macrophages generate excessive inflammatory cytokines IL-1*β* and IL-18 in a caspase-1-dependent manner [71]. In addition, in LPS-induced macrophages, knockout of the autophagic genes LC3-II and Beclin1 facilitates the secretion of IL-1*β* and IL-18, as well as the accumulation of mitochondrial ROS, which is dependent on the activation of the NLRP3 inflammasome [72]. It should be noted that autophagy also regulates macrophage apoptosis and further affects inflammation in asthma. A recent study has revealed that the lncRNA TRPM2-AS can inhibit autophagy-mediated apoptosis in macrophages by suppressing the TRIM21 (tripartite motif containing 21, an E3 ubiquitin ligase)-induced TRPM2 ubiquitination and further decreasing ROS levels, thus reducing the release of cytokines including IL-1*β*, IL-4, IL-6, IL-10, TNF-*α*, and TGF-*β* in asthma [73]. These findings indicate that repression of macrophage autophagy promotes the inflammatory responses in asthma, which are associated with mitochondrial ROS accumulation.

## 6. Advances, Limitations, and Perspectives

**Figure 3:**
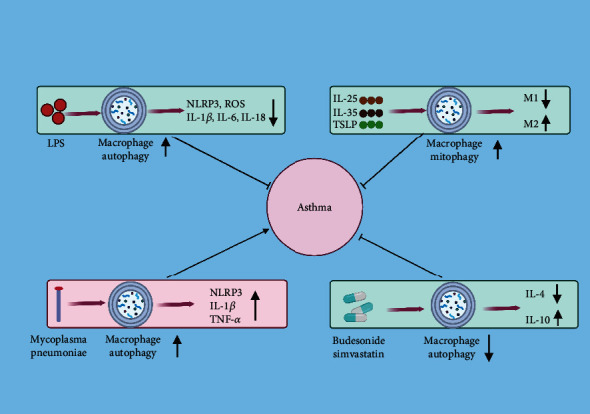
Role of macrophage autophagy in asthma. Macrophage autophagy regulates the progression of asthma by affecting the macrophage polarization and the release of inflammatory mediators. NLRP: NOD-like receptor protein.

Currently, macrophage autophagy is regarded as a crucial part of the host immune response, affecting disease progression and prognosis. Following activation, macrophage autophagy regulates and orchestrates its functions in inflammation and immunity based on different stimuli. However, whether macrophage autophagy plays a protective or a harmful role in asthma remains controversial. As shown in Figure 2, macrophage autophagy is implicated in the progression of asthma through several signaling pathways, such as MAPK, TLR4, and mTOR, regulating the polarization, inflammatory responses, and cell death. Consequently, the modulation of macrophage autophagy may provide promising therapeutic strategies for this disease (Figure 3). However, several limitations should be acknowledged when investigating the role of macrophage autophagy in asthma, and future studies are demanded to address these issues. First, although the functions and mechanisms of macrophage autophagy in asthma are increasingly investigated in the recent studies, the specific regulatory mechanisms remain unclear because of limited studies. Second, macrophage autophagy is modulated by multiple signaling pathways, and there are complex regulatory networks among these pathways that affect inflammatory responses and disease development in asthma, causing great difficulties in elucidating the role of autophagy in this disease. Third, macrophages are a very heterogeneous class of cells with highly dynamic phenotypes and functions; there are many types of macrophages in tissues, and various types of macrophages play different roles in asthma progression, and future studies should clarify whether autophagy has distinct roles in the polarization of different types of macrophages. In addition, it is noteworthy that the present research data are mainly based on animal models and in vitro experiments, and few autophagy regulators have been successfully applied in clinical practice. Owing to the functional heterogeneities of macrophages, when targeting macrophage autophagy at the gene or drug level for the treatment of asthma, the possible side effects should be fully considered. Besides, the tools for the accurate delivery of drugs to macrophages are demanded to be developed.

## 7. Conclusion and Perspective

In summary, macrophages act as a main component of innate immunity and play an important role in the progression of asthma, mediating both proinflammatory responses and anti-inflammatory functions; thus, regulating the function of macrophages may provide promising strategies for asthma treatment. Autophagy plays a protective role by regulating the inflammatory response and alleviating allergic airway inflammation and remodeling. Of interest, autophagy also performs a dual function in the development of asthma. How the autophagy mechanism exerts a paradoxical effect in this disease is elusive. In addition, macrophages initiate autophagy through signaling pathways such as MAPK, TLR4, and mTOR during asthmatic inflammation (Figure 2). Mitochondrial autophagy in macrophage promotes M2 phenotype polarization and plays an anti-inflammatory role, and repression of mitophagy contributes to ROS accumulation and aggravates inflammatory responses in asthma. However, macrophage autophagy in asthmatic patients with pathogen infection facilitates airway inflammation and asthma symptoms, and controlling excessive macrophage autophagy may play a protective role in asthma. In this context, the regulation of macrophage autophagy may become a potential therapeutic target for this disease (Figure 3). It should be noted that autophagy is a dynamic process that contains multiple stages, from the formation of autophagosomes to the fusion of autolysosomes. Thus, the mechanism by which the autophagic level of macrophages changes during different stages of asthma needs to be further explored, which provides a theoretical basis for developing novel interventions for this disease. In addition, macrophage autophagy is regulated by various signaling pathways, further identifying potential macrophage autophagy regulators such as epigenetic regulation of ATGs, and drugs targeting macrophage autophagy may have therapeutic potential in the treatment of asthma.

## Abbreviations

AMPK: Adenosine monophosphate-activated protein kinase
AMBRA: Autophagy and beclin-1 regulator
ATG: Autophagy-related gene
CCL: CC-motif chemokine ligand
IFN: Interferon
IL: Interleukin
LC3: Microtubule associated protein light chain 3
LPS: Lipopolysaccharide
mTOR: Mammalian target of rapamycin
MAPK: Mitogen activated kinase-like protein
NLRP: NOD-like receptor protein
PAS: Preautophagosomal structure
PE: Phosphatidylethanolamine
PI3K: Class III phosphatidylinositol 3-kinase
PI3P: Phosphatidylinositol 3-phosphate
PM: Particulate matter
ROS: Reactive oxygen species
TGF: Transforming growth factor
TLR: Toll-like receptor
TNF: Tumor necrosis factor
ULK1: Unc-51 like autophagy activating kinase
VPS15: vacuolar protein sorting 15
WIPI2: WD repeat domain phosphoinositide-interacting protein
ZFYVE1: Zinc-finger FYVE domain-containing protein 1.
